# Galectin-Glycan Interactions as Regulators of B Cell Immunity

**DOI:** 10.3389/fimmu.2018.02839

**Published:** 2018-12-04

**Authors:** Nicholas Giovannone, Logan K. Smith, Bebhinn Treanor, Charles J. Dimitroff

**Affiliations:** ^1^Department of Dermatology, Brigham and Women's Hospital, Boston, MA, United States; ^2^Harvard Medical School, Boston, MA, United States; ^3^Department of Immunology, University of Toronto, Toronto, ON, Canada; ^4^Department of Biological Sciences, University of Toronto Scarborough, Toronto, ON, Canada

**Keywords:** B cells, B cell activation, B cell receptor, galectin, I-branch, I-antigen, GCNT2

## Abstract

Cell surface glycans and their glycan-binding partners (lectins) have generally been recognized as adhesive assemblies with neighbor cells or matrix scaffolds in organs and the blood stream. However, our understanding of the roles for glycan-lectin interactions in immunity has expanded substantially to include regulation of nearly every stage of an immune response, from pathogen sensing to immune contraction. In this Mini-Review, we discuss the role of the ß-galactoside-binding lectins known as galectins specifically in the regulation of B-lymphocyte (B cell) development, activation, and differentiation. In particular, we highlight several recent studies revealing new roles for galectin (Gal)-9 in the modulation of B cell receptor-mediated signaling and activation in mouse and man. The roles for cell surface glycosylation, especially I-branching of N-glycans synthesized by the glycosyltransferase GCNT2, in the regulation of Gal-9 binding activity are also detailed. Finally, we consider how dysregulation of these factors may contribute to aberrant immune activation and autoimmune disease.

## Overview: Galectin-glycan Interactions in Immune Function

Galectins are a family of evolutionarily conserved glycan binding proteins (lectins) widely expressed in both stromal and immune tissues (1). In immunity, extensive research has established galectins as important regulators of immune homeostasis (2), inflammation (3), malignancy (4–6), and autoimmune disease (7). In the innate immune system, galectins are known to regulate granulocyte chemotaxis, dendritic cell maturation, mast cell activation, and many other activities (3). However, galectins are perhaps most widely recognized for their effects on T lymphocyte function, where galectins (Gal)-1,-3, and -9 have been shown to differentially modulate development, activation, differentiation, and effector function (3, 8, 9). The roles of galectins in innate and cell-mediated adaptive immunity have been reviewed at length elsewhere (1–4, 7, 8). Yet, while significant progress has been made in deciphering roles of galectins in innate immune cell and T cell biology, the roles for galectins in B cells have only recently begun to be deciphered. Here, we review the state of galectin literature in the B cell compartment, particularly with regard to B cell development, activation, differentiation, and effector function. We also discuss how differential glycosylation in B cells serves to regulate galectin function during different stages of B cell maturation. Finally, we conclude with emerging roles of galectins in B cell-mediated immune disease, particularly autoimmune disease.

## Galectins: Structure and Function

The glycan binding functions of galectins are mediated by highly conserved carbohydrate recognition domains (CRDs), which favor binding to β-galactoside-containing glycans, especially N-acetyllactosamines and their derivatives (1). To date, 15 galectins have been identified in mammals, classified based on their structure as either prototype, chimera-type, or tandem-repeat type (1). Prototype galectins, which includes galectins (Gal)-1,-2,-5,-7,-10,-11,-13,-14, and -15, possess one CRD and typically form homodimers by non-covalent association(1, 10–12). Chimera-type galectins, of which Gal-3 is the only family member identified to date, possess a single CRD connected to a collagen-like oligomerization domain that facilitates formation of higher order pentamers (13). Tandem-repeat type galectins include Gal-4,-8,-9, and -12 and contain two distinct CRDs that are covalently joined by a variable linker region (1, 14). The precise specificity of individual galectins varies somewhat between family members, and each galectin generally shows preference for a restricted set of glycoconjugates (1). Although galectins can function intracellularly, galectins predominantly operate at the cell surface and in endosomal compartments through interaction with membrane glycoconjugates (1, 15). Paradoxically, galectins lack a canonical secretion signal and therefore how galectins transit to the cell surface remains a major unresolved question in the field (1). Regardless of galectin structure and specificity, a unifying property is their capacity for bivalent or multivalent binding, which permits formation of galectin-glycoprotein networks called “lattices” that regulate glycoconjugate membrane dynamics (16–18). By tuning glycoprotein compartmentalization, diffusion speed, internalization, and lateral association with other glycoconjugates, galectins impact many critical cellular processes, especially signal transduction (16, 17). In some cases, the outcomes of galectin binding can be contradictory due to the vast diversity of galectin ligands, the binding of which can be modulated by the cell's metabolic, transcriptional, or glycosylation state (1). Therefore, the physiological functions of galectins are highly contextual, and consequently represent a dynamic mechanism to regulate immune cell activation and function.

## Galectins in B Cell Development

Galectins are recognized as regulators of thymic T cell development, with Gal-1,-3,-8, and -9 each reported to regulate thymocyte apoptosis and selection (19–25). However, a significant body of research has also amassed implicating galectins in early B cell development, particularly at the pre-BI to large pre-BII transition, when productively rearranged heavy chains pair with “surrogate” light chain (26) and the signaling chains CD79a/b (Igα/Igβ) form the pre-B cell receptor (pre-BCR) (26, 27). Signaling through the pre-BCR serves as a developmental checkpoint critical for pre-B cell expansion and development. However, whether signaling occurs by ligand-independent or ligand-dependent mechanisms has been a matter of debate (26).

Accumulating evidence suggests Gal-1 may serve ligand-like properties, albeit non-essential, in pre-BCR signaling. Using a combination of pulldown assays with recombinant surrogate light chain and surface plasmon resonance, Gauthier et al. identified that Gal-1 binds to the λ5 component of the surrogate light chain (28). In this and subsequent functional studies, Gal-1 was found to be produced by specialized bone marrow stromal cells that interact with pre-B cells, augmenting pre-BCR signaling by enhancing pre-BCR clustering at the pre-B cell/stromal cell synapse (28–33). Unusually, while Gal-1-mediated clustering of pre-BCR unequivocally depends on interactions between Gal-1 and several glycosylated pre-B cell integrins, binding of Gal-1 to surrogate light chain is not glycan-dependent (28, 31, 33). Instead, Gal-1 was found to interact with the “unique region” of λ5 via non-glycan-mediated hydrophobic interactions (34). Taken together, a model has emerged in which bone marrow stromal cell-secreted Gal-1 binds pre-B cell glycans expressed on integrins and facilitates pre-B cell / stromal cell synapse formation, while non-CRD-mediated interactions between Gal-1 and surrogate light chain subsequently promote pre-BCR clustering and signaling. However, it should be noted that the overall significance of Gal-1 to B cell development *in vivo* remains somewhat unresolved, as B cell development is minimally impaired in Gal-1-deficient mice (26, 30). How Gal-1 may overlap with other regulators of pre-BCR signaling, including heparan sulfates (35, 36), as well as with ligand-independent mechanisms of pre-BCR signaling, remains to be conclusively determined. Current paradigms suggest that both Gal-1-dependent and Gal-1-independent mechanisms jointly contribute to efficient pre-BCR signaling, and may exert compensatory activity (26).

Besides Gal-1, Gal-3 has also been implicated as a potential regulator of bone marrow B cell development. *LGALS3-/-* mice exhibit abnormal levels of several developing B cell subsets, including CD19+ B220+ c-Kit+ IL-7R+ pro-B cells (37). Accordingly, Gal-3-deficiency also correlated with dramatically augmented production of IL-7 transcript and increased levels of Notch ligands Jagged-1 and Delta-like 1 by bone marrow stroma in *LGALS3-/-* mice (37). While the precise mechanism was not investigated, these data suggest Gal-3 may act on bone marrow stroma to shape B cell development.

## Galectins in B Cell Signaling and Activation

In addition to the growing body of literature implicating a role for galectins in B cell development, emerging evidence suggests that galectins play important roles in the regulation of B cell signaling and activation. To date, Gal-1,-3, and-9 have each been implicated as both positive and/or negative regulators of B cell signaling.

In a recent study, Tsai et al. found that Gal-1 induces stimulatory signaling in murine B cells that bears hallmarks of antigen-receptor signaling through the BCR. They found that Gal-1 induces calcium flux, upregulation of B cell activation markers CD69 and CD86, and proliferation (38). Furthermore, using a phospho-proteomic approach, the authors observed that activation by Gal-1 leads to similar phosphorylation circuits as stimulation through IgM. Studies analyzing the role of Gal-1 *in vivo* revealed impaired proliferation of Gal-1-deficient B cells in response to antigenic challenge. Interestingly, Gal-1 from non-B cell sources was required for optimal B cell activation, as Gal-1 sufficient B cells in Gal-1 deficient hosts also showed reduced proliferation *in vivo*. Importantly, however, several groups have also reported that although Gal-1 is not highly expressed in resting mature B cells, it is highly upregulated with B cell activation, making the relevant contribution of B cell-intrinsic vs. B cell-extrinsic Gal-1 uncertain (39–42). In studies of B cell chronic lymphocytic leukemia (B-CLL) which depend on BCR signaling for survival and proliferation, Croci et al. observed that specialized tumor-supporting monocytes, so called “nurse-like” cells, enhanced BCR signaling and survival through the production of Gal-1 (43). Specifically, the authors found that Gal-1 bound B-CLL cells in a glycan-dependent manner and lowered the threshold of productive BCR signaling. Gal-1 also simultaneously promoted B-CLL survival through Gal-1-mediated enhancement of BAFF and APRIL expression by nurse-like cells. Collectively, these findings suggest a model where exogenous, and possibly B cell-intrinsic Gal-1, promote B cell activation through a BCR-dependent mechanism.

Paradoxically, however, in a few studies, Gal-1 has also been implicated as a negative regulator of B cell activation. In a study by Tabrizi et al. Gal-1 was highly expressed by resting and especially activated IgM+ memory B cells, inhibited Akt signaling, and promoted B cell death (40). Another study of human BL36 Burkitt lymphoma cells found that Gal-1 directly bound CD45 and inhibited its phosphatase activity (44). In mammalian two hybrid studies from the Roeder laboratory, Gal-1 was also found to bind (in a non-glycan-dependent mechanism) the B cell transcriptional co-activator and promoter of BCR signaling Oca-B, which the authors hypothesized inhibited cytoplasmic Gal-1 secretion and prevented Gal-1 induced suppression of CD45 phosphatase activity (41). Thus, the physiological functions for Gal-1 in B cells may be diverse, complex, and context dependent (44).

Besides Gal-1, many studies have implicated Gal-3 in the regulation of B cell activation. In a recent study by Beccaria et al. Gal-3 was also found to modulate B cell activation and germinal center (GC) immune responses. Specifically, the authors observed that Gal-3 was expressed in resting splenic B cells at steady state, and loss of Gal-3 in *LGALS3-/-* mice resulted in heightened activation (measured by CD80 and CD86 expression), spontaneous GC formation, augmented antibody secreting cell numbers, and increased circulating IgG2c and IgG3 (45). This phenotype was B cell-intrinsic, as adoptive transfer of *LGALS3-/-* B cells into B-cell deficient (but otherwise Gal-3-sufficient) mice showed similar results, as well as in other corroborating studies with *LGALS3-/-* B cells *in vitro*. Although the effects of Gal-3 were B cell-intrinsic, interplay between GC B cells and follicular T helper cells was postulated to be important, and IFNγ (produced most prominently by T cells but also B cells) was essential for spontaneous B cell GC formation. Additionally, data from several other studies of *LGALS3-/-* mice seem to support the overall conclusions of Beccaria et al., with *LGALS3-/-* showing overall improved antibody responses in several models of parasite infection, including *Plasmodium yoelii* (46) and *Schistosoma mansoni* infection models (37, 45, 47–50), but not *Plasmodium berghei* and *Plasmodium chabaudi* infection (46). Although a clear understanding of the molecular mechanisms involved is still lacking, studies of the role of Gal-3 in human diffuse large B cell lymphoma cell lines have shown that Gal-3 binds CD45, dampens its phosphatase activity, and promotes lymphoma cell survival (51). Interestingly, Gal-3 is known to be downregulated in primary human GC B cells (52), suggesting that loss of Gal-3 may be important for altering CD45 signaling activity within GCs, where CD45 is known to be essential for GC persistence (53). Additional studies will be required to decipher the molecular mechanisms operating that may restrict B cell activation.

In addition to Gal-3, Gal-9 has recently emerged as a negative regulator of BCR signaling and activation. Gal-9 was first implicated in the regulation of B cell activation in studies analyzing Gal-9-deficient mice, where Sharma et al. observed that mice lacking Gal-9 have increased viral-specific IgM, IgG, and IgA titers as well as enhanced formation of antibody secreting cells in response to influenza A challenge (54). These initial data were further supported by studies in human B cells, which demonstrated that recombinant and mesenchymal stem cell-derived Gal-9 antagonizes B cell proliferation and antibody-secreting cell formation in a dose dependent manner, and that treatment of mice with recombinant Gal-9 *in vivo* resulted in diminished antigen specific serum titers in response to immunization (55).

**Figure 1:**
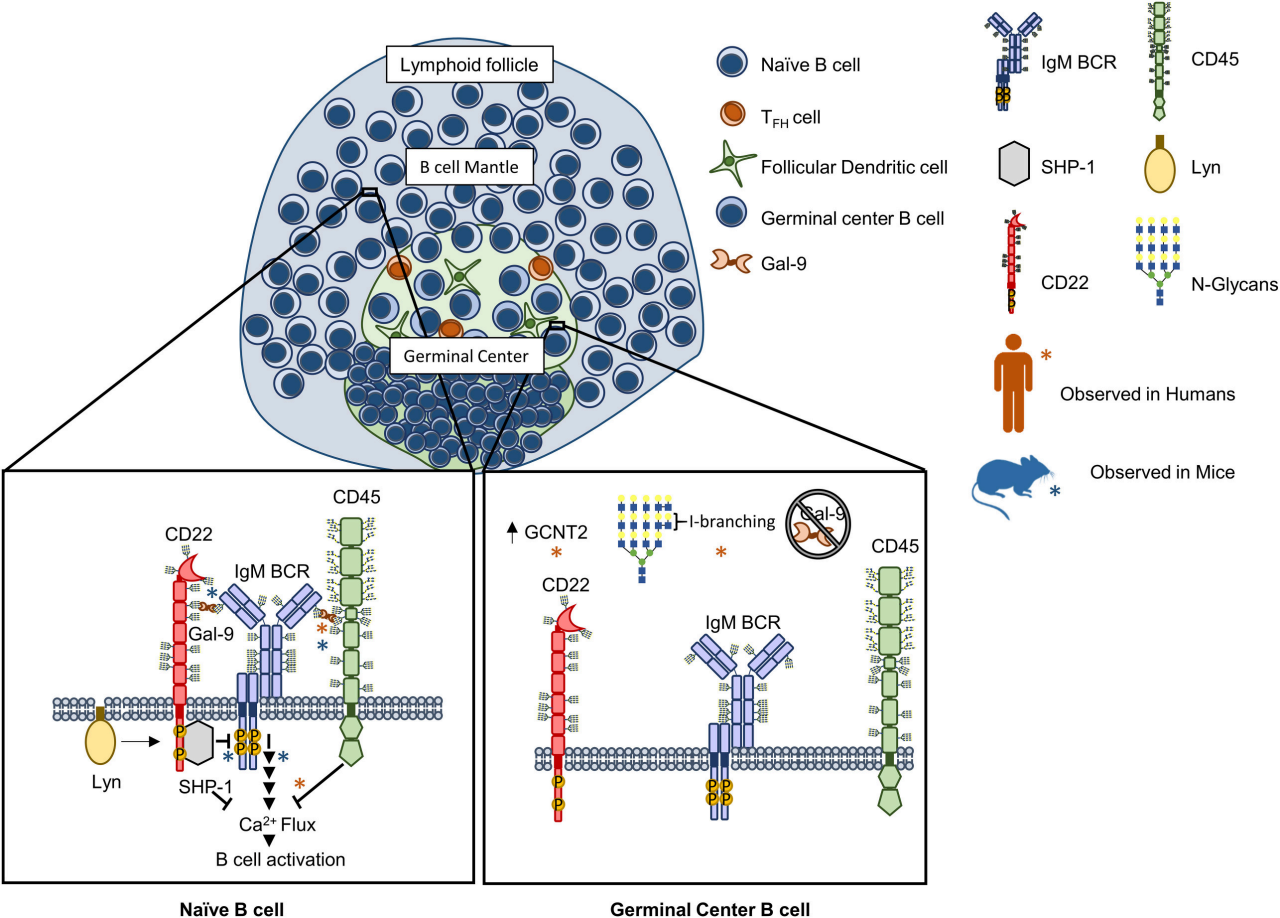
Galectin-9 regulates B cell receptor signaling in human and murine B cells through analogous but distinct mechanisms. Naïve B cell activation is antagonized by Gal-9 through binding to the receptor tyrosine phosphatase CD45 on human B cells that activates a Lyn-CD22-SHP-1 dependent circuit and inhibits calcium accumulation downstream of the BCR [Left, orange asterisks (*)]. In murine B cells, Gal-9 similarly regulates BCR signaling by altering the nanoscale organization of signaling molecules. Specifically, Gal-9 has been found to bind CD45 but also IgM BCR, preventing exclusion of CD45 and CD22 upon B cell activation and leading to impaired signal transduction following BCR ligation [Left, blue asterisks (*)]. In humans, Gal-9 binding activity is differentially regulated between naïve and germinal center (GC) B cells via concerted alterations in N-glycosylation. Specifically, Gal-9 binding has been shown to be greatly diminished in germinal center (GC) B cells via upregulation of the glycosyltransferase GCNT2, which catalyzes I-branch formation on glycan ligands of Gal-9 (poly-LacNAcs), and attenuates Gal-9 binding (56, 57).

Recently, our groups independently investigated the molecular mechanisms for Gal-9 mediated regulation of B cell activation (56, 57). We found that Gal-9 is detectable on the surface of primary naïve B cells in both mice and humans and could act in a B cell-intrinsic manner to negatively regulate BCR signaling. Mechanistically, Gal-9 antagonized BCR signal transduction by similar but slightly different mechanisms. In human B cells, we found that a major Gal-9 receptor was CD45 (57). Binding of CD45 by Gal-9 triggered a negative signaling cascade through Lyn, CD22, and SHP-1 that dampened BCR-triggered calcium flux and inhibited activation of calcium-sensitive transcription factors, including NFAT-1 and NF-κB. In an analogous but distinct manner, in murine B cells, we observed that Gal-9 bound not only CD45 but also IgM-BCR (56). Functionally, murine Gal-9 regulated BCR-antigen microclustering and downstream signaling, both of which were enhanced in Gal-9-deficient murine B cells. However, rather than altering calcium signaling, murine Gal-9 mitigated activation of CD19 and ERK1/2 downstream of BCR ligation. We hypothesize that this impaired signaling response is due to Gal-9's ability to prevent exclusion of inhibitory receptors from the signalosome, as we found CD45 and CD22 are specifically enriched within Gal-9 lattices and showed enhanced colocalization with IgM-BCR. Moreover, using dual-color super-resolution microscopy, we observed that association of IgM-BCR with CD22 is reduced in resting Gal-9-deficient B cells, and propose that this provides a plausible mechanism for enhanced BCR signaling in the absence of Gal-9 (56). Taken together these data suggest that Gal-9 acts to attenuate BCR signaling through facilitating interactions with endogenous regulatory networks (Figure 1). These findings provide exciting potential for therapeutic development targeting steady-state B cell signaling networks.

## Galectins as Modulators of B Cell Differentiation and Cell Fate

An accumulating body of evidence suggests that galectins can also influence cell fate decisions in mature B cells, particularly in regulating the balance of B cell differentiation to memory or plasma cells.

Acosta-Rodriguez et al. examined the role of Gal-3 in B cells both *in vitro* and *in vivo* following *Trypanosoma cruzi* infection (58). The authors found that Gal-3 was upregulated in response to IL-4 or CD40-mediated stimulation and in B cells during ongoing parasite infection *in vivo*. Silencing of Gal-3 by RNA interference *in vitro* and *in vivo* prevented IL-4-induced downregulation of Blimp-1, a transcription factor critical for plasma cell development, and enhanced plasma cell differentiation. A mechanism was proposed in which Gal-3 works in concert with IL-4 to disfavor plasma cell differentiation and promote differentiation to memory B cells (58). Indeed, this hypothesis has since been supported by numerous studies demonstrating increased antibody-secreting cell numbers and antibody titers at steady state and in response to parasite infection in *LGALS3–/–* mice (37, 45–50). Interestingly, B-lymphopenia, significantly disrupted follicular architecture in lymph nodes and spleen, increased spontaneous GC numbers, and lupus-like pathology have also been reported for *LGALS3–/–* mice (45, 47, 48, 50).

In contrast to Gal-3, data suggests that Gal-1 and Gal-8 favor plasma cell fate decisions. Studies examining Gal-1 expression in murine and human B cells have noted that Gal-1 is significantly upregulated with B cell differentiation and is directly induced by Blimp-1 (39, 59). Through a combination of *in vitro* approaches that included ectopic expression, genetic knockdown, synthetic galectin inhibitors, and use of galectin-deficient mice, Tsai et al. demonstrated Gal-1 is sufficient to positively regulate plasma cell differentiation *in vitro* (39, 59). However, the authors propose that Gal-1 is not strictly required, as Gal-8 was found to be able to functionally compensate for loss of Gal-1 (39). In a separate study, however, Anginot et al. demonstrate that, at least *in vivo*, Gal-1 is required for optimal plasma cell responses and may not be fully compensated by Gal-8 (60). Specifically, Gal-1-deficient mice exhibited impaired antibody secreting cell number and diminished IgM and IgG titers in response to immunization, particularly in response to the T-dependent antigens (60). Interestingly, both groups report that Gal-1 is produced by (39, 59, 60) and binds (39, 59) only early plasma cells and not fully differentiated plasma cells, suggesting that Gal-1 and -8 drive the earliest stages of plasma cell differentiation. While the specific mechanism of action remains unresolved, Gal-1 and Gal-8 expression and/or treatment were associated with enhanced expression of XBP-1 (Gal-1 and Gal-8), Blimp-1 (Gal-8), IL-10 (Gal-1), and IL-6 (Gal-8) (39). In addition, Gal-1 also appears to have pro-survival roles in plasma cells (60). By contrast, Gal-1 has been reported to be expressed by IgM+ memory B cells, in which it was shown to inhibit Akt signaling compared to Gal-1^lo^ naïve B cells and promote BCR-induced apoptosis (40). Thus, Gal-1/-8 and Gal-3 appear to have opposing roles in skewing the outcome of B cell differentiation.

## Galectins in B Cell Effector Function

Galectins have also been reported to have roles in the regulation of B cell effector function. As secreted molecules, galectins can exert cytokine-like activity. A documented example is Gal-1, which is upregulated with B cell activation and secreted into the B cell milieu, where it has been shown to induce apoptosis of inflammatory T cells (42). Besides serving as effector cytokines, galectins have also been reported to augment the immunoglobulin secretion capacity of plasma cells. Although Gal-1 and Gal-8 facilitate plasma cell differentiation (described earlier), they also have been shown to directly enhance secretion of antibody by augmenting expression of XBP-1s and by increasing the ratio of secreted / membrane IgM transcripts (39, 59). Once secreted, antibodies themselves can be bound by galectins. Both Gal-3 and Gal-9 have been shown to bind IgE bound to mast cell FcεR and prevent clustering-induced degranulation of inflammatory mediators (61–63).

## Glycosylation in the Regulation of Galectin Activity

As lectins, extracellular galectin activity is often highly dependent on the favorable glycosylation of target receptors (1). Receptor glycosylation is regulated by a host of factors, including cell metabolism, ER and Golgi nucleotide sugar-donor transporters, and the rate of glycoprotein flux through the Golgi (64). Frequently, however, receptor glycosylation is dictated by the expression and activity of relevant ER- and Golgi-resident glycosyltransferases, glycosidases, and glycan-modifying enzymes.

Recently, our laboratory analyzed the global N-glycan repertoire of human tonsillar naïve, GC, and memory B cells by whole glycome mass spectrometry (MS) and plant lectin based flow cytometry (57). We found that all three B cell subsets expressed tri- and tetra-antennary complex-type N-glycans replete with poly-N-acetyllactosamine (poly-LacNAc), which are repeating units of the disaccharide N-acetyllactosamine (Gal-GlcNAc) that canonically serve as high affinity binding determinants for many galectins. Indeed, poly-LacNAc expression by naïve and memory B cells corresponded with robust binding to Gal-1 and, to our surprise, Gal-9, which had previously not been reported to bind B cells. However, whereas Gal-1 showed similarly strong binding to GC B cells as non-GC cell types, Gal-9 binding was starkly reduced in GC B cells. Closer examination of the N-glycomic profile of naïve, GC, and memory B cells by tandem MS revealed that many poly-LacNAcs in GC B cells were modified with internal β1,6 GlcNAc- and galactose-containing disaccharides, termed “I-branches” or I-blood group antigen, that were not present in naïve and memory B cells (57). I-branch expression at the GC stage corresponded with upregulated expression of the I-branching enzyme, GCNT2, and genetic studies in B cell lines demonstrated that GCNT2/I-branches were both necessary and sufficient to inhibit Gal-9 binding (57), as well as Gal-3 binding [(65) and unpublished observations] (Figure 1). Interestingly, Gal-1 binding was largely unaffected by I-branches, suggesting that I-branches may preferentially target Gal-3 and Gal-9 glycan binding motifs, whereas terminal modifications such as α2,6-sialylation by the sialyltransferase ST6Gal1 may more selectively target Gal-1 (1, 8). Therefore, selective glycosyltransferase expression may be a mechanism of disparately regulating the activity of different galectin family members in B cells.

Beyond I-branches, regulation of Core 2 poly-LacNAc expression on O-glycans by GCNT1 was shown by Clark et al. to be a major factor controlling Gal-3 binding in B cell lines (51). Whereas B cell lines expressing GCNT1 showed robust binding and cell surface localization of Gal-3, B cells with inherently low GCNT1 or GCNT1 knockdown did not (51). Although the expression pattern of GCNT1 in native B cell populations was not determined, studies in our laboratory (66) indicate that naïve and GC B cells robustly express GCNT1/Core 2 poly-LacNAcs, whereas more differentiated B cells (memory B cells and plasmablasts) downregulate GCNT1/Core 2 poly-LacNAcs. The significance of this glycan expression pattern to Gal-3 binding activity is currently under investigation by our laboratory.

It is important to emphasize that while glycosylation can significantly contribute to the regulation of galectin activity, not all galectin functionality is exclusively glycan-dependent. In a study by Bonzi et al., it was demonstrated that non-glycosylation dependent interactions between Gal-1 and pre-BCR induce conformational changes in the Gal-1 CRD in a manner that alter its glycan-binding preferences, perhaps allowing Gal-1 to disengage from glycosylated integrin ligands and promote non-glycosylation dependent pre-BCR clustering. Moreover, galectins have been shown to exhibit roles intracellularly in mitochondrial, cytoplasmic, and nuclear compartments (15).

As novel functions emerge for galectins in B cells, identifying the factors regulating their activity, especially expression of favorable glycan ligands, will remain crucial to understanding their physiological role *in vivo*, and how galectin-glycan interactions may be exploited therapeutically.

## Galectins in B Cell Autoimmunity

The increasingly apparent roles for galectins in modulating B cell activation and cell fate suggests that galectins may serve important roles as regulators of B cell tolerance. While clearly a budding and ongoing area of investigation, a few studies have suggested potential (albeit complex) roles for Gal-1, Gal-3, and Gal-9 in B cell-mediated autoimmune disease (67). Recent evidence suggests that Gal-3 deficient mice develop systemic autoimmunity with lupus-like features, including spontaneous GC formation, elevated levels of anti-nuclear antibodies, and kidney pathology (45). This lupus-like pathology became increasingly pronounced with age, and was found to be absolutely dependent on B cell-intrinsic Gal-3 as well as IFNγ, the production of which was increased in both B cells and T cells (68). Of note, studies by Clark et al. also noted autoantibody development in *LGALS3–/–* mice when crossed to LamH transgenic mice, which express an antibody heavy chain reactive against the self-antigen laminin (69). Interestingly, the numbers of autoreactive B cells that escape tolerance mechanisms are increased further in mice doubly deficient for both Gal-3 and Gal-1 (69). While specific mechanisms linking Gal-3, and possibly Gal-1, to maintaining B cell tolerance have yet to be fully elucidated, it should be noted that both Gal-3 and Gal-1 have been shown to be more highly expressed in anergic murine B cells (68).

Besides Gal-1 and Gal-3, Gal-9 has also been implicated in the development of lupus-like disease in several models of SLE. In a recent study, Panda *et al*. demonstrated that treatment of BXSB/MpJ and (NZB × NZW)F1 lupus-prone mice with Gal-9 before symptoms manifests diminishes the probability of developing pathology, including tissue inflammation and splenomegaly associated with disease onset (70). Mechanistically, the authors present evidence that Gal-9 antagonizes TLR7- and TLR9-dependent activation of plasmacytoid dendritic cells (pDCs) and B cells, as well as type I Interferon production by pDCs. In a second study from Moritoki et al., Gal-9 was found to ameliorate pathology in a MRL/lpr model of lupus, apparently by inducing plasma cell apoptosis, although a direct link was not firmly established (71). Interestingly, these two studies are seemingly opposite to findings from Zeggar et al., who using a pristane-induced lupus model, observed that *LGALS9–/–* mice exhibited reduced disease burden and unaltered TLR7-type I interferon signaling (72). The reasons underlying these disparate results are unclear, but may reflect the different model systems used, including spontaneous *vs*. inducible models of lupus and disparate moue backgrounds (73). Future studies will be required to parse the precise contribution of Gal-9, Gal-3, and Gal-1 to B cell tolerance, and to better determine a possible role for these lectins (or relevant glycans) in the development of autoimmune disease in humans.

## Conclusions and Outlook

**Figure 2:**
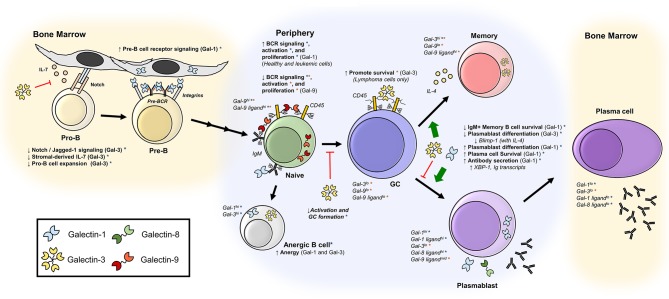
Galectins regulate B cell development, activation, and differentiation. Depicted are published roles of galectins at various stages of differentiation, as well as reported expression of galectins and galectin-binding glycans. Orange asterisks (*) indicate findings described in human B cells, and blue asterisks (*) indicate findings observed in mice. Bone marrow B cells (28–34, 37, 60); naïve B cells (38, 43, 45, 56, 57); anergic B cells (37, 68); GC B cells (45, 51); memory B cells (37, 40, 45–50, 58); plasmablasts and plasma cells (37, 39, 59, 60).

Here, we have reviewed the emerging roles of galectins in B cell immunobiology. Over the past two decades, studies have revealed a complex network of positive and negative regulatory roles for galectins acting throughout B cell development, activation, differentiation, and antibody responses (Figure 2). Recent studies in particular have highlighted novel roles for Gal-9 and glycosylation in the regulation of BCR signaling and activation. Moving forward, studies investigating the precise mechanisms of galectin function in B cells, and concomitant regulation of galectin activity by B cell glycosylation, will be of great interest. Furthermore, how galectins contribute to B cell-mediated disease, including autoimmune disease, will remain a critical area of future research that will likely yield important insights into disease etiology and/or novel therapeutic approaches targeting galectin-glycan interactions. Undoubtedly, the continued investigation of the multitudinous and complex roles of galectins in B cell biology will be an exciting pursuit in the years ahead.

## Author Contributions

All authors listed have made a substantial, direct and intellectual contribution to the work, and approved it for publication.

### Conflict of Interest Statement

The authors declare that the research was conducted in the absence of any commercial or financial relationships that could be construed as a potential conflict of interest.

## References

[1] Rabinovich GA Toscano MA. Turning ‘sweet’ on immunity: galectin-glycan interactions in immune tolerance and inflammation. Nat Rev Immunol. (2009) 9:338–52. 10.1038/nri2536 19365409

[2] Rabinovich GA Croci DO. Regulatory circuits mediated by lectin-glycan interactions in autoimmunity and cancer. Immunity (2012) 36:322–35. 10.1016/j.immuni.2012.03.004 22444630

[3] Liu FT Rabinovich GA. Galectins: regulators of acute and chronic inflammation. Ann N Y Acad Sci. (2010) 1183:158–82. 10.1111/j.1749-6632.2009.05131.x 20146714

[4] Rabinovich GA Conejo-Garcia JR. Shaping the immune landscape in cancer by galectin-driven regulatory pathways. J Mol Biol. (2016) 428:3266–81. 10.1016/j.jmb.2016.03.021 27038510

[5] Dimitroff CJ. Galectin-binding O-glycosylations as regulators of malignancy. Cancer Res. (2015) 75:3195–202. 10.1158/0008-5472.CAN-15-0834 26224120 PMC4537818

[6] Cedeno-Laurent F Dimitroff CJ. Galectins and their ligands: negative regulators of anti-tumor immunity. Glycoconj J. (2012) 29:619–25. 10.1007/s10719-012-9379-0 22544342 PMC3410977

[7] Toscano MA Martinez Allo VC Cutine AM Rabinovich GA Marino KV. Untangling galectin-driven regulatory circuits in autoimmune inflammation. Trends Mol Med. (2018) 24:348–63. 10.1016/j.molmed.2018.02.008 29555188

[8] Cedeno-Laurent F Dimitroff CJ. Galectin-1 research in T cell immunity: past, present and future. Clin Immunol. (2012) 142:107–16. 10.1016/j.clim.2011.09.011 22019770 PMC3266984

[9] Perillo NL Pace KE Seilhamer JJ Baum LG. Apoptosis of T cells mediated by galectin-1. Nature (1995) 378:736–9. 10.1038/378736a0 7501023

[10] Cho M Cummings RD. Characterization of monomeric forms of galectin-1 generated by site-directed mutagenesis. Biochemistry (1996) 35:13081–8. 10.1021/bi961181d 8855944

[11] Cho M Cummings RD. Galectin-1, a beta-galactoside-binding lectin in Chinese hamster ovary cells. ILocalization I, and biosynthesis. J Biol Chem. (1995) 270:5207–12. 10.1074/jbc.270.10.5207 7890631

[12] Cho M Cummings RD. Galectin-1, a beta-galactoside-binding lectin in Chinese hamster ovary cells. Physical I, and chemical characterization. J Biol Chem. (1995) 270:5198–206. 10.1074/jbc.270.10.5198 7890630

[13] Ahmad N Gabius HJ Andre S Kaltner H Sabesan S Roy R. Galectin-3 precipitates as a pentamer with synthetic multivalent carbohydrates and forms heterogeneous cross-linked complexes. J Biol Chem. (2004) 279:10841–7. 10.1074/jbc.M312834200 14672941

[14] Wada J Kanwar YS. Identification and characterization of galectin-9, a novel beta-galactoside-binding mammalian lectin. J Biol Chem. (1997) 272:6078–86. 10.1074/jbc.272.9.6078 9038233

[15] Vladoiu MC Labrie M St-Pierre Y. Intracellular galectins in cancer cells: potential new targets for therapy (Review). Int J Oncol. (2014) 44:1001–14. 10.3892/ijo.2014.2267 24452506

[16] Nabi IR Shankar J Dennis JW. The galectin lattice at a glance. J Cell Sci. (2015) 128:2213–9. 10.1242/jcs.151159 26092931

[17] Rabinovich GA Toscano MA Jackson SS Vasta GR. Functions of cell surface galectin-glycoprotein lattices. Curr Opin Struct Biol. (2007) 17:513–20. 10.1016/j.sbi.2007.09.002 17950594 PMC2100406

[18] Brewer CF Miceli MC Baum LG. Clusters, bundles, arrays and lattices: novel mechanisms for lectin-saccharide-mediated cellular interactions. Curr Opin Struct Biol. (2002) 12:616–23. 10.1016/S0959-440X(02)00364-0 12464313

[19] Clark MC Baum LG. T cells modulate glycans on CD43 and CD45 during development and activation, signal regulation, and survival. Ann N Y Acad Sci. (2012) 1253:58–67. 10.1111/j.1749-6632.2011.06304.x 22288421 PMC4190024

[20] Bi S Earl LA Jacobs L Baum LG. Structural features of galectin-9 and galectin-1 that determine distinct T cell death pathways. J Biol Chem. (2008) 283:12248–58. 10.1074/jbc.M800523200 18258591 PMC2431002

[21] Tribulatti MV Mucci J Cattaneo V Aguero F Gilmartin T Head SR. Galectin-8 induces apoptosis in the CD4(high)CD8(high) thymocyte subpopulation. Glycobiology (2007) 17:1404–12. 10.1093/glycob/cwm104 17893094

[22] Stillman BN Hsu DK Pang M Brewer CF Johnson P Liu FT. Galectin-3 and galectin-1 bind distinct cell surface glycoprotein receptors to induce T cell death. J Immunol. (2006) 176:778–89. 10.4049/jimmunol.176.2.778 16393961

[23] Hernandez JD Nguyen JT He J Wang W Ardman B Green JM. Galectin-1 binds different CD43 glycoforms to cluster CD43 and regulate T cell death. J Immunol. (2006) 177:5328–36. 10.4049/jimmunol.177.8.5328 17015718

[24] Villa-Verde DM Silva-Monteiro E Jasiulionis MG Farias-De-Oliveira DA Brentani RR Savino W. Galectin-3 modulates carbohydrate-dependent thymocyte interactions with the thymic microenvironment. Eur J Immunol. (2002) 32:1434–44. 10.1002/1521-4141(200205)32:5&lt;1434::AID-IMMU1434&gt;3.0.CO;2-M 11981832

[25] Perillo NL Uittenbogaart CH Nguyen JT Baum LG. Galectin-1, an endogenous lectin produced by thymic epithelial cells, induces apoptosis of human thymocytes. J Exp Med. (1997) 185:1851–8. 10.1084/jem.185.10.1851 9151710 PMC2196320

[26] Reth M Nielsen P. Signaling circuits in early B-cell development. Adv Immunol. (2014) 122:129–75. 10.1016/B978-0-12-800267-4.00004-3 24507157

[27] Ubelhart R Werner M Jumaa H. Assembly and function of the precursor B-cell receptor. Curr Top Microbiol Immunol. (2016) 393:3–25. 10.1007/82_2015_475 26415650

[28] Gauthier L Rossi B Roux F Termine E Schiff C. Galectin-1 is a stromal cell ligand of the pre-B cell receptor (BCR) implicated in synapse formation between pre-B and stromal cells and in pre-BCR triggering. Proc Natl Acad Sci USA. (2002) 99:13014–9. 10.1073/pnas.202323999 12271131 PMC130578

[29] Erasmus MF Matlawska-Wasowska K Kinjyo I Mahajan A Winter SS Xu L. Dynamic pre-BCR homodimers fine-tune autonomous survival signals in B cell precursor acute lymphoblastic leukemia. Sci Signal (2016) 9:ra116. 10.1126/scisignal.aaf3949 27899526 PMC5385842

[30] Espeli M Mancini SJ Breton C Poirier F Schiff C. Impaired B-cell development at the pre-BII-cell stage in galectin-1-deficient mice due to inefficient pre-BII/stromal cell interactions. Blood (2009) 113:5878–86. 10.1182/blood-2009-01-198465 19329777

[31] Rossi B Espeli M Schiff C Gauthier L. Clustering of pre-B cell integrins induces galectin-1-dependent pre-B cell receptor relocalization and activation. J Immunol. (2006) 177:796–803. 10.4049/jimmunol.177.2.796 16818733

[32] Mourcin F Breton C Tellier J Narang P Chasson L Jorquera A. Galectin-1-expressing stromal cells constitute a specific niche for pre-BII cell development in mouse bone marrow. Blood (2011) 117:6552–61. 10.1182/blood-2010-12-323113 21511956

[33] Bonzi J Bornet O Betzi S Kasper BT Mahal LK Mancini SJ. Pre-B cell receptor binding to galectin-1 modifies galectin-1/carbohydrate affinity to modulate specific galectin-1/glycan lattice interactions. Nat Commu. (2015) 6:6194. 10.1038/ncomms7194 25708191

[34] Elantak L Espeli M Boned A Bornet O Bonzi J Gauthier L. Structural basis for galectin-1-dependent pre-B cell receptor (pre-BCR) activation. J Biol Chem. (2012) 287:44703–13. 10.1074/jbc.M112.395152 23124203 PMC3531785

[35] Bradl H Wittmann J Milius D Vettermann C Jack HM. Interaction of murine precursor B cell receptor with stroma cells is controlled by the unique tail of lambda 5 and stroma cell-associated heparan sulfate. J Immunol. (2003) 171:2338–48. 10.4049/jimmunol.171.5.2338 12928380

[36] Vettermann C Herrmann K Albert C Roth E Bosl MR Jack HM. A unique role for the lambda5 nonimmunoglobulin tail in early B lymphocyte development. J Immunol. (2008) 181:3232–42. 10.4049/jimmunol.181.5.3232 18713994

[37] de Oliveira FL Dos Santos SN Ricon L da Costa TP Pereira JX Brand C El-Cheikh MC, Lack of galectin-3 modifies differentially Notch ligands in bone marrow and spleen stromal cells interfering with B cell differentiation. Sci Rep. (2018) 8:3495 10.1038/s41598-018-21409-7 29472568 PMC5823902

[38] Tsai CM Wu HY Su TH Kuo CW Huang HW Chung CH. Phosphoproteomic analyses reveal that galectin-1 augments the dynamics of B-cell receptor signaling. J Proteomics (2014) 103:241–53. 10.1016/j.jprot.2014.03.031 24704852

[39] Tsai CM Guan CH Hsieh HW Hsu TL Tu Z Wu KJ. Galectin-1 and galectin-8 have redundant roles in promoting plasma cell formation. J Immunol. (2011) 187:1643–52. 10.4049/jimmunol.1100297 21753146

[40] Tabrizi SJ Niiro H Masui M Yoshimoto G Iino T Kikushige Y. T Cell Leukemia/Lymphoma 1 and galectin-1 regulate survival/cell death pathways in human naive and IgM+ memory B cells through altering balances in Bcl-2 family proteins. J Immunol. (2009) 182:1490–9. 10.4049/jimmunol.182.3.1490 19155496

[41] Yu X Siegel R Roeder RG. Interaction of the B cell-specific transcriptional coactivator OCA-B and galectin-1 and a possible role in regulating BCR-mediated B cell proliferation. J Biol Chem. (2006) 281:15505–16. 10.1074/jbc.M509041200 16565088

[42] Zuniga E Rabinovich GA Iglesias MM Gruppi A. Regulated expression of galectin-1 during B-cell activation and implications for T-cell apoptosis. J Leukoc Biol. (2001) 70:73–9. 10.1189/jlb.70.1.73 11435488

[43] Croci DO Morande PE Dergan-Dylon S Borge M Toscano MA Stupirski JC. Nurse-like cells control the activity of chronic lymphocytic leukemia B cells via galectin-1. Leukemia (2013) 27:1413–6. 10.1038/leu.2012.315 23257714

[44] Fouillit M Joubert-Caron R Poirier F Bourin P Monostori E Levi-Strauss M. Regulation of CD45-induced signaling by galectin-1 in Burkitt lymphoma B cells. Glycobiology (2000) 10:413–9. 10.1093/glycob/10.4.413 10764829

[45] Beccaria CG Amezcua Vesely MC Fiocca Vernengo F Gehrau RC Ramello MC Tosello Boari J. Galectin-3 deficiency drives lupus-like disease by promoting spontaneous germinal centers formation via IFN-gamma. Nat Commun. (2018) 9:1628. 10.1038/s41467-018-04063-5 29691398 PMC5915532

[46] Toscano MA Tongren JE de Souza JB Liu FT Riley EM Rabinovich GA. Endogenous galectin-3 controls experimental malaria in a species-specific manner. Parasite Immunol. (2012) 34:383–7. 10.1111/j.1365-3024.2012.01366.x 22486577

[47] Brand C Oliveira FL Takiya CM Palumbo A Jr Hsu DK Liu FT. The involvement of the spleen during chronic phase of Schistosoma mansoni infection in galectin-3-/- mice. Histol Histopathol. (2012) 27:1109–20. 10.14670/HH-27.1109 22763883

[48] Oliveira FL Brand C Paula AA Arcanjo KD Hsu DK Liu FT. Lack of galectin-3 disturbs mesenteric lymph node homeostasis and B cell niches in the course of Schistosoma mansoni infection. PLoS ONE (2011) 6:e19216. 10.1371/journal.pone.0019216 21573150 PMC3089595

[49] Oliveira FL Chammas R Ricon L Fermino ML Bernardes ES Hsu DK. Galectin-3 regulates peritoneal B1-cell differentiation into plasma cells. Glycobiology (2009) 19:1248–58. 10.1093/glycob/cwp120 19696234

[50] Oliveira FL Frazao P Chammas R Hsu DK Liu FT Borojevic R. Kinetics of mobilization and differentiation of lymphohematopoietic cells during experimental murine schistosomiasis in galectin-3 -/- mice. J Leukoc Biol. (2007) 82:300–10. 10.1189/jlb.1206747 17456800

[51] Clark MC Pang M Hsu DK Liu FT de Vos S Gascoyne RD. Galectin-3 binds to CD45 on diffuse large B-cell lymphoma cells to regulate susceptibility to cell death. Blood (2012) 120:4635–44. 10.1182/blood-2012-06-438234 23065155 PMC3512238

[52] Hoyer KK Pang M Gui D Shintaku IP Kuwabara I Liu FT. An anti-apoptotic role for galectin-3 in diffuse large B-cell lymphomas. Am J Pathol. (2004) 164:893–902. 10.1016/S0002-9440(10)63177-X 14982843 PMC1614710

[53] Huntington ND Xu Y Puthalakath H Light A Willis SN Strasser A. CD45 links the B cell receptor with cell survival and is required for the persistence of germinal centers. Nat Immunol. (2006) 7:190–8. 10.1038/ni1292 16378097

[54] Sharma S Sundararajan A Suryawanshi A Kumar N Veiga-Parga T Kuchroo VK. T cell immunoglobulin and mucin protein-3 (Tim-3)/Galectin-9 interaction regulates influenza A virus-specific humoral and CD8 T-cell responses. Proc Natl Acad Sci USA (2011) 108:19001–6. 10.1073/pnas.1107087108 22052881 PMC3223445

[55] Ungerer C Quade-Lyssy P Radeke HH Henschler R Konigs C Kohl U Seifried E. Galectin-9 is a suppressor of T and B cells and predicts the immune modulatory potential of mesenchymal stromal cell preparations. Stem Cells Dev. (2014) 23:755–66. 10.1089/scd.2013.0335 24083426 PMC3967371

[56] Cao A Alluqmani N F.Buhari HM Wasim L Smith LK Quaile AT. Galectin-9 binds IgM-BCR to regulate B cell signaling. Nat Commun. (2018) 9:3288. 10.1038/s41467-018-05771-8 30120235 PMC6098130

[57] Giovannone N Liang J Antonopoulos A Geddes Sweeney J King SL Pochebit SM. Galectin-9 suppresses B cell receptor signaling and is regulated by I-branching of N-glycans. Nat Commun. (2018) 9:3287. 10.1038/s41467-018-05770-9 30120234 PMC6098069

[58] Acosta-Rodriguez EV Montes CL Motran CC Zuniga EI Liu FT Rabinovich GA. Galectin-3 mediates IL-4-induced survival and differentiation of B cells: functional cross-talk and implications during Trypanosoma cruzi infection. J Immunol. (2004) 172:493–502. 10.4049/jimmunol.172.1.493 14688359

[59] Tsai CM Chiu YK Hsu TL Lin IY Hsieh SL Lin KI. Galectin-1 promotes immunoglobulin production during plasma cell differentiation. J Immunol. (2008) 181:4570–9. 10.4049/jimmunol.181.7.4570 18802059

[60] Anginot A Espeli M Chasson L Mancini SJ Schiff C. Galectin 1 modulates plasma cell homeostasis and regulates the humoral immune response. J Immunol. (2013) 190:5526–33. 10.4049/jimmunol.1201885 23616571 PMC3660186

[61] Niki T Tsutsui S Hirose S Aradono S Sugimoto Y Takeshita K. Galectin-9 is a high affinity IgE-binding lectin with anti-allergic effect by blocking IgE-antigen complex formation. J Biol Chem. (2009) 284:32344–52. 10.1074/jbc.M109.035196 19776007 PMC2781649

[62] Zuberi RI Frigeri LG Liu FT. Activation of rat basophilic leukemia cells by epsilon BP, an IgE-binding endogenous lectin. Cell Immunol. (1994) 156:1–12. 10.1006/cimm.1994.1148 8200029

[63] Frigeri LG Zuberi RI Liu FT. Epsilon BP, a beta-galactoside-binding animal lectin, recognizes IgE receptor (Fc epsilon RI) and activates mast cells. Biochemistry (1993) 32:7644–9. 10.1021/bi00081a007 8347574

[64] Marth JD Grewal PK. Mammalian glycosylation in immunity. Nat Rev Immunol. (2008) 8:874–87. 10.1038/nri2417 18846099 PMC2768770

[65] Sweeney JG Liang J Antonopoulos A Giovannone N Kang S Mondala TS. Loss of GCNT2/I-branched glycans enhances melanoma growth and survival. Nat Commun. (2018) 9:3368. 10.1038/s41467-018-05795-0 30135430 PMC6105653

[66] Giovannone N Antonopoulos A iang J Geddes Sweeney J Kudelka MR King SL Human B Cell Differentiation is characterized by progressive remodeling of o-linked glycans. Front. Immunol. (2018) 9:2857 10.3389/fimmu.2018.02857 PMC6302748 30619255

[67] Rhee I Veillette A. Protein tyrosine phosphatases in lymphocyte activation and autoimmunity. Nat Immunol. (2012) 13:439–47. 10.1038/ni.2246 22513334

[68] Clark AG Chen S Zhang H Brady GF Ungewitter EK Bradley JK. Multifunctional regulators of cell growth are differentially expressed in anergic murine B cells. Mol Immunol. (2007) 44:1274–85. 10.1016/j.molimm.2006.06.001 16890292

[69] Clark AG Weston ML Foster MH. Lack of galectin-1 or galectin-3 alters B cell deletion and anergy in an autoantibody transgene model. Glycobiology (2013) 23:893–903. 10.1093/glycob/cwt026 23550149 PMC3671777

[70] Panda SK Facchinetti V Voynova E Hanabuchi S Karnell JL Hanna RN. Galectin-9 inhibits TLR7-mediated autoimmunity in murine lupus models. J Clin Invest. (2018) 128:1873–87. 10.1172/JCI97333 29611821 PMC5919878

[71] Moritoki M Kadowaki T Niki T Nakano D Soma G Mori H. Galectin-9 ameliorates clinical severity of MRL/lpr lupus-prone mice by inducing plasma cell apoptosis independently of Tim-3. PLoS ONE (2013) 8:e60807. 10.1371/journal.pone.0060807 23585851 PMC3621869

[72] Zeggar S Watanabe KS Teshigawara S Hiramatsu S Katsuyama T Katsuyama E. Role of Lgals9 deficiency in attenuating nephritis and arthritis in BALB/c mice in a pristane-induced lupus model. Arthritis Rheumatol. (2018) 70:1089–101. 10.1002/art.40467 29481735

[73] Panda AK Das BK. Perplexing role of galectin 9 in experimental lupus models: comment on the article by zeggar et al. Arthritis Rheumatol. (2018) 70:1530–1. 10.1002/art.40564 29781131

